# General and Central Obesity Are Associated With Increased Severity of the VMS and Sexual Symptoms of Menopause Among Chinese Women: A Longitudinal Study

**DOI:** 10.3389/fendo.2022.814872

**Published:** 2022-04-26

**Authors:** Ruiyi Tang, Yubo Fan, Min Luo, Duoduo Zhang, Zhuolin Xie, Feiling Huang, Yuchen Wang, Gaifen Liu, Yaping Wang, Shouqing Lin, Rong Chen

**Affiliations:** ^1^ Department of Obstetrics and Gynecology, Peking Union Medical College Hospital, Chinese Academy of Medical Science and Peking Union Medical College, Beijing, China; ^2^ National Clinical Research Center for Obstetric and Gynecologic Diseases, Beijing, China; ^3^ Department of Neurology, Beijing Tiantan Hospital, Capital Medical University, Beijing, China

**Keywords:** obesity, menopause, abdominal obesity, menopausal symptoms, vasomotor symptoms, mood symptoms

## Abstract

**Background:**

Strong evidence has linked overweight and obesity to increased risks of cardiovascular disease and all-cause mortality in Chinese populations. Menopause is considered associated with increased obesity and central body fat distribution. However, the correlation between obesity and menopausal symptoms has not been well studied.

**Objective:**

To examine the associations between obesity or abdominal obesity and menopausal symptoms as women progressed from premenopausal to postmenopausal status.

**Design:**

This study included 430 midlife Chinese women who had experienced natural menopause and were followed up for 10 years. Physical examinations and questionnaires should be completed annually. The questionnaires include the Menopause-Specific Quality of Life questionnaire, the Hospital Anxiety and Depression Scale, and other physical and behavioral factors.

**Results:**

Among women who were not obese (n=345) or not abdominal obese (n=372) at baseline, 5.8% and 31.7% became obese or abdominal obese at the recent follow-up visit, respectively. Women at the recent follow-up visit had an increased body mass index (BMI) by 0.14%, and the waist-to-hip ratio (WHR) increased by 5.2% compared with the data at baseline. In multivariate analysis, more frequent hot flashes, moderate/severe bothered vasomotor symptoms (VMS), mild bothered sexual functioning, and less anxiety symptoms were significantly associated with obesity. Increasing age, moderate/severe bothered VMS, and less anxiety symptoms were independently associated with abdominal obesity. Multivariable analysis also showed that less education level is independently associated with both obesity and abdominal obesity.

**Conclusion:**

Our findings suggest that the proportion of obesity and abdominal obesity increased gradually during menopause. The increase of abdominal obesity is more rapidly than obesity in middle-aged women. Both obesity and abdominal obesity are related with severe or frequent VMS and anxiety symptoms in Chinese women. Although the proportion of obese women in China is lower than in western countries, the problem of abdominal obesity and related complications cannot be ignored.

## Introduction

Obesity is one of the most pressing threats to public health because its increasing prevalence globally and its significance as a major risk factor of a variety of chronic conditions (1, 2). Strong evidence has linked overweight and obesity to increased risks of major non-communicable diseases and premature mortality in Chinese populations (3). Women experience higher rates of obesity than men. The prevalence of obesity increases in women during menopausal transition (4), which may be linked to the increase in cardiovascular events observed following the menopausal transition. Previous studies have shown that mid-life obesity is associated with a more symptomatic menopausal transition, including greater reporting of vasomotor symptoms (VMS, including hot flashes and sweats), hormone dynamics (5, 6), previous menstrual cycle length (7), among others. Mood dysfunction is widespread among menopausal women, and a recent study showed that it is associated with VMS, fatigue, and change of body composition or obesity (8). Weight status is considered as a key effector of menopausal and mood symptoms in menopausal women (9, 10).

Furthermore, the menopause is also considered to be associated with increased central body fat distribution. Fat distribution can be assessed crudely using waist circumference, hip circumference and the waist-to-hip ratio (WHR) (11). Some evidence suggests that increasing obesity during midlife is related with chronological aging whereas changes in body composition and fat distribution are primarily due to ovarian aging (12).

With increasing life expectancy, almost every woman would experience the transition to menopause, and one third of a woman’s lifetime would be spent in postmenopausal stage. It is of great significance to appropriately manage menopause, especially menopausal symptoms and chronic degenerative diseases. Understanding the relationship between menopausal symptoms and obesity may help identify new strategies to prevent weight gain in women at midlife, then to reduce the incidence of complications. Therefore, related study has important clinical significance in promoting women’s health.

The incidence rate of obesity varies by races (13). African-American and Hispanic women progressed more quickly to obesity, whereas Chinese and Japanese women progressed more slowly (13). However, accumulated evidence also showed that Chinese people are likely to have higher percentages of body fat and higher rates of cardiovascular risk factors and all-cause mortality than White people at given body mass index (BMI) levels (14–16). Then, the risk factors of obesity in menopausal women may also vary by races. According to the Chinese people’s situation, China has formulated a diagnostic standard for obesity in Chinese (BMI≥28) (17), different from the diagnostic criteria of obesity in other countries (BMI≥30) (18). In the past decades, the rapid economic growth in China has been accompanied by alarming rise of obese populations (3). A population-based study conducted in northern China showed that 30.2% of female aged 18–79 years were overweight and 12.8% were obese (19).

Based on previous studies, we hypothesize that obesity increases the severity of menopausal symptoms. This study aims to examine associations between obesity and menopausal symptoms as women progressing from premenopausal to postmenopausal status. We used data from Peking Union Medical College Hospital Aging Longitudinal Cohort of Women in Midlife (PALM), a longitudinal study of women in midlife in China.

## Materials And Methods

### Participants

Data are collected from the PALM study, a prospective, community-based longitudinal cohort in Beijing, China, and described in previous reports (20). This study was initiated in July 2005. Since baseline, participants have been followed up annually. A trained, qualified nurse from the Department at Gynecology in Peking Union Medical College Hospital (PUMCH) managed the baseline and follow-up interviews and arranged for participants to complete the laboratory tests.

At enrollment in the cohort, the ages of the participants were 35 to 64 years old, all participants had an intact uterus and at least one ovary, and were not pregnant or breastfeeding. Exclusion criteria for cohort enrollment included histories of severe systemic diseases, reproductive endocrinologic disorders, use of hormonal medications in the previous 3 months, including hormonal contraception and hormone treatment. More complete information on screening and data collection has been published previously (20). Ethical approval was granted by the institutional review board of the PUMCH (JS-2100), and all participants provided written informed consents.

For the full cohort, 954 women were enrolled. In the present analysis, a sub-cohort of 430 women were included in this study. To be eligible for this study, each woman had to have completed at least two obesity, VMS, and the Hospital Anxiety and Depression Scale (HADS) assessments during annual follow-up visits, and had an observable final menstrual period (FMP) followed by at least 1 year of amenorrhea during the follow-up. Women who had a hysterectomy after enrollment were excluded. This study included data from all the annual follow-up visits of the 430 women in the sub-cohort. We primarily analyzed the relationship between obesity and menopausal stages, as well as menopausal symptoms, focusing on the VMS and mood symptoms. The BMI data and VMS was evaluated at each assessment, whereas the HADS (21) was introduced since 2006 to evaluate depressive and anxiety symptoms. In this analysis, the visit at which the participant had their first HADS assessment was regarded as the baseline visit.

### Measures

#### Measures of Obesity

Obesity was assessed by anthropometric examinations. Height and weight were measured at each assessment period with participants in light clothing and without shoes, and calibrated scales were used. BMI was calculated as weight in kilograms divided by height in meters squared, and was classified according to Chinese-specific criteria (17) as follows: non-obese (<24 kg/m^2^), overweight (24–27.9 kg/m^2^), and obesity (≥28 kg/m^2^). Waist circumference and hip circumference were also recorded annually. WHR, which was calculated as waist circumference divided by hip circumference, is a measure of visceral fat. Abdominal obesity was defined as WHR ≥0.85.

#### Assessment of Menopausal Symptoms

At each follow-up, participants were asked about the presence, frequency, and severity of hot flashes in the prior two weeks. The women should answer the question: “How often did you experience hot flashes in the past 2 weeks” (Never, 1–2 times/day, 3–9 times/day, ≥10times/day)?

In addition, the Menopause-Specific Quality of Life (MenQol) questionnaire (22) was used to evaluate the bother of menopausal symptoms. The MenQol questionnaire consists of 27 items in four domains: vasomotor (items 1–3), psychosocial (items 4–10), physical (items 11–24), and sexual functioning (items 25–27). Women reported whether they had experienced each symptom in the previous month. The participants were asked whether they had experienced the symptoms in the previous month (range from 1 – “not experiencing symptoms or feeling” to 8 – “extremely bothered”). Each domain score was calculated as the mean of the item scores. The severity of the bother of symptoms in each domain was evaluated by the average domain score, and was classified as none (1), mild (>1 and ≤5), or moderate/severe (>5) (23, 24).

Symptoms of anxiety and depression were evaluated by the Chinese version of HADS (21, 25–27). The HADS is a self-reported scale consisting of 14 items (range from 0 – “no distress” to 3 – “the highest level of distress”). The anxiety subscale (, 7 items) enquires about worry and restlessness, whereas the depression subscale (HADS-D, 7 items) focuses on depressed mood, lack of enjoyment, and hopelessness. An average score of the HADS-A or HADS-D ≥8 was taken to indicate the presence of anxiety or depressive symptoms (28).

#### Assessment of Menopausal Status

The definitions of menopausal stage were from the 2011 Stages of Reproductive Aging Workshop+10 (STRAW+10) criteria (29) as follows: (1) premenopause (regular menstrual periods in the last 3 months and no change in menstrual frequency in the last 12 months); (2) early perimenopausal transition (two or more cycles with a difference in cycle length of >7 d); (3) late perimenopausal transition (amenorrhea interval of >60 d); and (4) postmenopause (the period after 12 consecutive months of amenorrhea). Postmenopause were divided into Stage +1a (<1 year after FMP), Stage +1b (1 to 2 years after FMP), Stage +1c (2 to 8 years after FMP) and Stage +2 (>8 years after FMP).

#### Covariates

Age, menopausal age, level of education, marital status, income, and general health status was also included in this analysis. General health status was based on self-report and was classified as poor, good, or excellent.

### Statistical Analysis

Continuous variables with normal distributions were expressed as mean ± standard deviation (SD). Categorical variables were presented as percentages. Comparisons of continuous variables were made using the t-test and Mann–Whitney U-test for normal and non-normal data, respectively. Categorical data were compared using the chi-squared test, and ordered categorical data were compared using the non-parametric Kruskal-Wallis test.

At baseline, three BMI groups (non-obese/overweight/obese) and two WHR groups (<0.85 and ≥0.85) were compared regarding baseline characteristics using t-test, chi-square or Kruskal-Wallis tests. This study is a longitudinal cohort study with repeated measurements from the same subjects over time. We used binary logistic or ordered logistic generalized estimating equations (GEE) (30) to account for within-participant correlations through a working correlation matrix. This strategy permitted within-subject correlations and missing visits. It enabled researchers to accurately estimate the effect size in case of incomplete data, and could provide comparable results to those of other repeated-measure analyses done on complete datasets. We estimated the relationship between degree of obesity or abdominal obesity and the menopausal status, menopausal symptoms, and other potential related factors. The models included a random intercept only. An exchangeable working correlation matrix that accounts for correlations within subjects was used.

Covariates that were considered clinically relevant or that had previously been reported to be associated with the outcomes were separately added to the basic model. Candidate variables with a P value ≤ 0.2 in the univariate analyses were included in multivariable model to assess the independent effects of these variables on the outcomes. Both univariate and multivariable analysis were carried-out using GEE.

Analyses were performed using SPSS software (version 24.0, IBM). All tests were two-sided with a 0.05 significance level.

## Results

### Participants’ Baseline Characteristics

Over the 10-year follow-up period, the 430 women had a total of 2,533 assessments, with two to eight visits each (mean 5.9), and 46.9% were retained in the cohort in 2015. **Table 1** describes the baseline characteristics of our cohort according to their baseline obesity status and abdominal obesity status. At baseline, 19.8% of the 430 women were classified as obesity, while 13.5% as having abdominal obesity. Among women who were not obese (n=345) at baseline, during the follow-up, 20 women became obese at the recent follow-up visit, and the incidence of obesity was 5.8%. For women who were not abdominal obese (n=372) at baseline, 118 women (31.7%) became abdominal obese at the recent follow-up visit. When compared the data of the recent follow-up visit with the data at baseline, the BMI increased by 0.14%, the weight decreased by 0.91%. The WHR increased by 5.2% whereas the waist circumference increased by 4.2%. In **Table 1**, the risk of obesity was significantly higher in women who were older and had more severe VMS and less education. Women with abdominal obesity were significantly older and had higher income.

**Table 1:** Baseline sample description of all participants according to the presence of obesity and abdominal obesity.

Characteristic	All participants	Non-obesity (BMI < 24) (N = 160; 37.2%)	Overweight (BMI ≥ 24 and <24) (N = 185; 43.0%)	Obesity (BMI≥28) (N = 85; 19.8%)	P_1_	No abdominal obesity (WHR < 0.85) (N = 372;86.5%)	Abdominal obesity (WHR ≥ 0.85) (N = 58;13.5%)	P_2_
**N**	430	160	185	85		372	58	
**Age, mean (SD), yr**	52.5 (6.4)	50.9 (6.3)	53.1 (5.9)	53.4 (7.0)	**0.002**	52.1 (6.3)	53.9 (6.2)	**0.04**
**Menopausal age, mean (SD), yr**	50.4 (3.2)	50.2 (3.2)	50.6 (3.2)	50.3 (3.3)	0.42	49.7 (3.5)	50.1 (3.9)	0.65
**BMI, mean (SD), kg/m^2^**	25.3 (3.4)	22.0 (1.4)	25.9 (1.1)	30.2 (2.1)	**<0.001**	25.1 (3.2)	26.9 (3.9)	**<0.001**
**Currently Smoking, No. (%)**	3 (0.7)	1 (0.6)	2 (1.1)	0 (0.0)	0.46	2 (0.5)	1 (1.7)	0.38
**Presence of anxiety symptoms, No. (%)**	40 (9.3)	19 (11.9)	17 (9.2)	4 (4.7)	0.16	37 (9.9)	3 (5.2)	0.33
**Presence of depressive symptoms, No. (%)**	96 (22.3)	26 (16.3)	49 (26.5)	21 (24.7)	0.06	87 (23.4)	9 (15.5)	0.23
**Hot flashes, No. (%)**					0.08			0.51
None	269 (62.6)	103 (64.4)	113 (61.1)	53 (62.4)		234 (62.9)	35 (60.3)	
≤2/d	122 (28.4)	50 (31.3)	51 (27.6)	21 (24.7)		107 (28.8)	15 (25.9)	
3-9/d	33 (7.7)	7 (4.4)	18 (9.7)	8 (9.4)		27 (7.3)	6 (10.3)	
≥10/d	6 (1.4)	0 (0.0)	3 (1.6)	3 (3.5)		4 (1.1)	2 (3.4)	
**Severity of VMS, No. (%)**					**0.02**			0.08
Not bothered	232 (54.0)	83 (51.9)	99 (53.5)	50 (58.8)		205 (55.1)	27 (46.6)	
Mild bothered	188 (43.7)	77 (48.1)	80 (43.2)	31 (36.5)		161 (43.3)	27 (46.6)	
Moderate/severe bothered	10 (2.3)	0 (0)	6 (3.2)	4 (4.7)		46(1.6)	4 (6.9)	
**Severity of psychological symptoms, No. (%)**					0.95			0.38
Not bothered	40 (9.3)	15 (9.4)	16 (8.6)	9 (10.6)		33 (8.9)	7 (12.1)	
Mild bothered	381 (88.6)	141 (88.1)	165 (89.2)	75 (88.2)		330 (88.7)	51 (87.9)	
Moderate/severe bothered	9 (2.1)	4 (2.5)	4 (2.2)	1 (1.2)		9 (2.4)	0	
**Severity of physical symptoms, No. (%)**					0.13			0.61
Not bothered	10 (2.4)	6 (3.8)	3 (1.6)	1 (1.2)		9 (2.4)	1 (1.8)	
Mild bothered	401 (94.4)	150 (94.3)	170 (92.9)	81 (97.6)		350 (94.6)	51 (92.7)	
Moderate/severe bothered	14 (3.3)	3 (1.9)	10 (5.5)	1 (1.2)		11 (3.0)	3 (5.5)	
**Severity of sexual functioning symptoms, No. (%)**					0.41			0.11
Not bothered	131 (30.7)	54 (34.0)	48 (26.1)	29 (34.5)		116 (31.4)	15 (26.3)	
Mild bothered	206 (48.2)	71 (44.7)	98 (53.3)	37 (44.0)		182 (49.2)	24 (42.1)	
Moderate/severe bothered	90 (21.1)	34 (21.4)	38 (20.7)	18 (21.4)		72 (19.5)	18 (31.6)	
**Menopausal status, No. (%)**					0.37			0.38
Premenopausal	70 (16.3)	34 (21.3)	26 (14.1)	10 (11.8)		60 (16.1)	10 (17.2)	
Early menopausal transition	55 (12.8)	24 (15.0)	22 (11.9)	9 (10.6)		52 (14.0)	3 (5.2)	
Late menopausal transition	65 (15.1)	28 (17.5)	26 (14.1)	11 (12.9)		56 (15.1)	9 (15.5)	
Postmenopause, Stage +1a	21 (4.9)	6 (3.8)	9 (4.9)	6 (7.1)		17 (4.6)	4 (6.9)	
Postmenopause, Stage +1b	22 (5.1)	7 (4.4)	10 (5.4)	5 (5.9)		19 (5.1)	3 (5.2)	
Postmenopause, Stage +1c	111 (25.8)	37 (23.1)	53 (28.6)	21 (24.7)		98 (26.3)	13 (22.4)	
Postmenopause, Stage +2	86 (20.0)	24 (15.0)	39 (21.1)	23 (27.1)		70 (18.8)	16 (27.6)	
**Income, No. (%)**					0.61			**0.03**
<1000RMB	200 (46.6)	69 (43.1)	88 (47.6)	43 (51.2)		177 (47.7)	23 (39.7)	
1000-2000 RMB	153 (35.7)	58 (36.3)	65 (35.1)	30 (35.7)		136 (36.7)	17 (29.3)	
>2000 RMB	76 (17.7)	33 (20.6)	32 (17.3)	11 (13.1)		58 (15.6)	18 (31.0)	
**General health status, No. (%)**					0.75			0.71
excellent	85 (19.9)	31.(19.5)	41 (22.2)	13 (15.5)		73 (19.7)	12 (21.1)	
good	322 (75.2)	120 (74.8)	136 (73.5)	67 (79.8)		281 (75.7)	41 (71.9)	
poor	21 (4.9)	9 (5.7)	8 (4.3)	4 (4.8)		17 (4.6)	4 (7.0)	
**Marital status, No. (%)**					0.71			
Single	3 (0.7)	1 (0.6)	1 (0.5)	1 (1.2)		2 (0.5)	1 (1.7)	0.11
Married	391 (90.9)	147 (91.9)	170 (91.9)	74 (87.1)		336 (90.3)	55 (94.8)	
Widowed	17 (4.0)	4 (2.5)	7 (3.8)	6 (7.1)		17 (0.5)	0 (1.7)	
Divorced	19 (4.4)	8 (5.0)	7 (3.8)	4 (4.7)		17 (4.6)	2 (3.4)	
**Educational status, No. (%)**					**0.004**			0.88
Middle school	131 (30.5)	32 (20.0)	62 (33.5)	37 (44.0)		113 (30.5)	18 (31.0)	
High school	152 (35.4)	62 (38.8)	61 (33.0)	29 (34.5)		131 (35.3)	21 (36.2)	
College	113 (26.3)	49 (30.6)	50 (27.0)	14 (16.7)		97 (26.1)	16 (27.6)	
University or higher	33 (7.7)	17 (10.6)	12 (6.5)	4 (4.8)		30 (8.1)	3 (5.2)	

BMI, body mass index; WHR, waist-to-hip ratio; VMS, vasomotor symptom; SD, standard deviation; RMB, Renminbi.

The bold values indicated P < 0.05.

### Obesity/Abdominal Obesity and Menopausal Status

**Figure 1:**
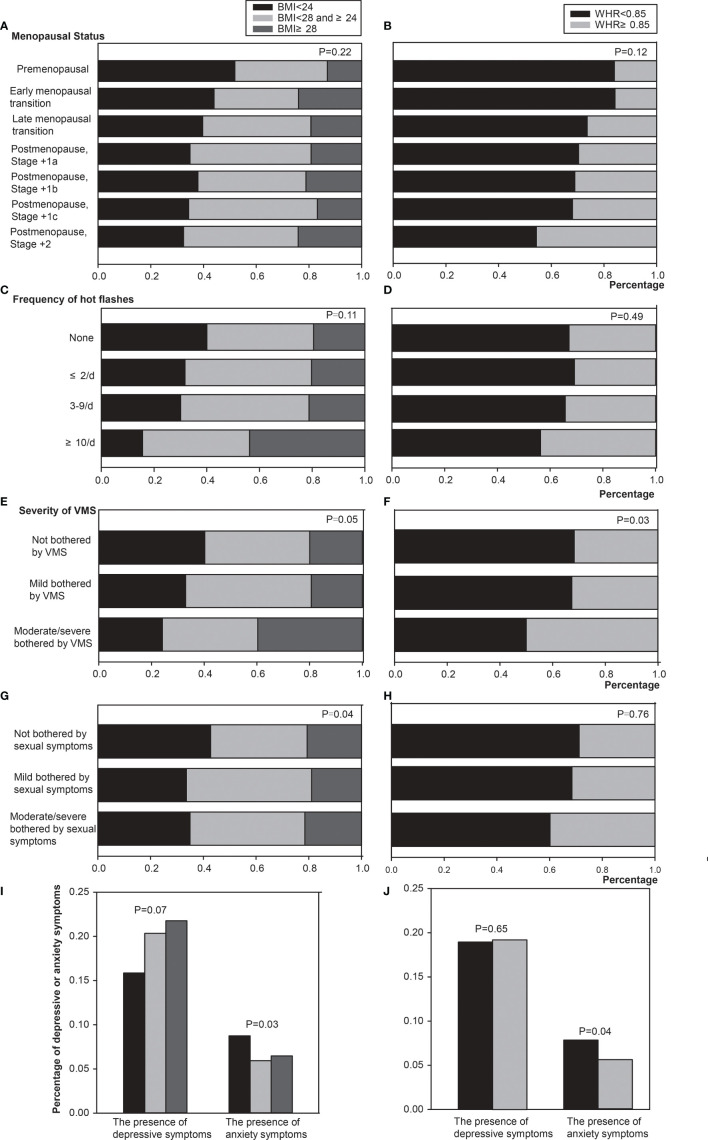
Relationship between obesity or abdominal obesity and menopausal symptoms. The P values were estimated by generalized estimating equations used for repeated measures. **(A)** The relationship between menopausal stage and obesity. **(B)** The relationship between menopausal stage and abdominal obesity. **(C)** The relationship between frequency of hot flashes and obesity. **(D)** The relationship between frequency of hot flashes and abdominal obesity. **(E)** The relationship between severity of VMS and obesity. **(F)** The relationship between severity of VMS and abdominal obesity. **(G)** The relationship between sexual symptoms and obesity. **(H)** The relationship between sexual symptoms and abdominal obesity. **(I)** The relationship between depressive or anxiety symptoms and obesity. **(J)** The relationship between depressive or anxiety symptoms and abdominal obesity. BMI, body mass index; WHR, waist-to-hip ratio; VMS, vasomotor symptom.

**Figures 1A, B** described the relationship between menopausal stages and obesity or abdominal obesity. The risk of obesity/abdominal obesity seems to increase with advancing menopausal stages. **Table 2** showed the results of multivariate analysis with obesity or abdominal obesity as dependent variables respectively. In multivariate analysis, the risk of obesity in perimenopausal women (including menopausal transition and postmenopausal +1a stage) was significantly higher than that in premenopausal women. Postmenopausal women (+1b, +1c and +2 stage) had a significantly higher rate of abdominal obesity than premenopausal women.

**Table 2:** Multivariate associations of risk factors with obesity and abdominal obesity during 10 years follow-up.

Variables	Multivariable Model of Obesity		Multivariable Model of Abdominal Obesity	
	P_1_ Value	OR (95%CI)	P_2_ Value	OR (95%CI)
**Age, years**	0.320	1.01 (0.99-1.03)	**0.003**	1.08 (1.03-1.13)
**Presence of anxiety symptoms**	**0.03**	0.72 (0.53-0.97)	**0.04**	0.70 (0.49-0.99)
**Presence of depressive symptoms**	0.07	1.13 (0.99-1.28)	0.65	1.06 (0.83-1.35)
**Hot flashes**	0.11		0.49	
None		Reference		Reference
≤2/d		1.08 (0.93-1.25)		0.80 (0.61-1.06)
3-9/d		0.98 (0.83-1.16)		0.84 (0.57-1.24)
≥10/d		1.30 (1.02-1.66)*		0.94 (0.45-1.95)
**Severity of VMS**	**0.05**		**0.03**	
Not bothered		Reference		Reference
Mild bothered		1.04 (0.92-1.17)		1.25 (0.96-1.63)
Moderate/severe bothered		1.35 (1.05-1.75)*		2.41 (1.26-4.61)**
**Severity of psychological symptoms**			0.14	
Not bothered				Reference
Mild bothered				0.82 (0.58-1.20)
Moderate/severe bothered				1.29 (0.67-2.48)
**Severity of sexual functioning symptoms**	**0.04**		0.76	
Not bothered		Reference		Reference
Mild bothered		1.13 (1.01-1.27)*		1.08 (0.87-1.34)
Moderate/severe bothered		1.02 (0.87-1.19)		1.07 (0.82-1.39)
**Menopausal status**	0.22		0.12	
Premenopausal		Reference		Reference
Early menopausal transition		1.36 (1.02-1.82)*		1.06 (0.47-2.41)
Late menopausal transition		1.40 (1.05-1.87)*		2.14 (0.98-4.65)
Postmenopause, Stage +1a		1.43 (1.02-2.02)*		2.12 (0.91-4.93)
Postmenopause, Stage +1b		1.37 (0.95-1.98)		2.52 (1.05-6.06)*
Postmenopause, Stage +1c		1.29 (0.88-1.89)		2.45 (1.00-6.00)*
Postmenopause, Stage +2		1.35 (0.87-2.11)		3.12 (1.09-8.97)*
**Educational status**	**0.001**		**0.047**	
Middle school		Reference		Reference
High school		0.64 (0.43-0.94)*		1.48 (1.03-2.12)*
College		0.49 (0.33-0.72)***		1.01 (0.69-1.49)
University or higher		0.38 (0.19-0.77)**		0.63 (0.30-1.32)

*<0.05; **≤0.01; ***≤0.001.

BMI, body mass index; VMS, vasomotor symptom; RMB, Renminbi; OR, Odds Ratio; CI, confidential interval.

The bold values indicated P < 0.05.

### Obesity/Abdominal Obesity and MenQol Results

**Figures 1C–F** depicted the associations of obesity or abdominal obesity with VMS frequency and severity. In univariable analysis, women with more frequent or severe VMS were significantly more likely to be obese (P=0.02). The abdominal obesity was significantly correlated with the severity of VMS (P=0.03), but not the VMS frequency (P=0.34). The risk of obesity and abdominal obesity was significantly higher among women with≥10 times hot flashes a day and women evaluated as moderately/severely bothered by VMS. The relationships between obesity or abdominal obesity and psychosocial, physical, as well as sexual functioning symptoms were also evaluated. In univariable analysis, only sexual functioning symptoms were significantly associated with abdominal obesity (P=0.04).

In multivariate analysis showed in **Table 2**, there was no significant correlation between both obesity or abdominal obesity and the frequency of hot flashes. However, it seemed that women with ≥10 times hot flashes a day were more likely to be obese (P<0.05). The severity of VMS was significantly correlated with both obesity and abdominal obesity. The risk of abdominal obesity in women moderate/severe bothered by VMS was 2.41 times higher than those who were not (P<0.01). Psychosocial and sexual functioning symptoms were included in the multivariable model because of the P value ≤ 0.2 in the univariate analyses. Sexual functioning symptoms were independently associated with obesity (**Figure 1G**), and not associated with abdominal obesity (**Figure 1H**). Women with mildly bothering sexual functioning symptoms had a 1.13 times higher risk of obesity compared with women without sexual functioning symptoms (P=0.04).

### Obesity/Abdominal Obesity and Mood Symptoms

As shown in **Figures 1I, J**, obese women were more likely to have depressive symptoms and less likely to have anxiety symptoms. But the correlation between obesity and depressive symptoms was not statistically significant (P=0.14). The proportion of depressive symptoms in women accompanied by abdominal obesity seems to increase slightly. In the multivariable model, anxiety symptoms in women with obesity or abdominal obesity were significantly lower than those women without obesity or abdominal obesity (P<0.05). However, the correlation between abdominal obesity and depressive symptoms had no statistical significance (**Table 2**).

### Age and Education Status Are Independent Risk Factor of Abdominal Obesity

A correlation between menopausal age and obesity/abdominal obesity was not found. In addition to the variables mentioned above, results of multivariable analysis showed that education status was independently associated with obesity and abdominal obesity. Furthermore, after adjusting the menopausal stages, age was still an independent risk factor for abdominal obesity (**Table 2**).

## Discussion

This is the first prospective study of ovarian aging in Chinese women in midlife. The present analysis explored the correlation between menopausal symptoms and obesity, showing that both obesity and abdominal obesity were significantly correlated with decreased severity of anxiety symptoms and serious disturbing VMS, but there was no significant relationship between obesity and depressive symptoms. Obesity also increases the severity of sexual functioning symptoms.

Epidemiologic evidence suggests that the mid-life period is a critical window for increases in body weight and changes in body composition for women (12). Aging is associated with oxidative stress that promotes the accumulation of saturated ceramide and diacylglycerol fatty acids, increasing systemic fat and visceral adipose tissue (31, 32). Overweight and obesity are established risk factors for many metabolic diseases (33), which have become an increasingly public health problem. The risk of obesity increases in many women during menopausal transition and is significantly correlated with menopausal symptoms, which includes VMS, depressive symptoms, anxiety symptoms, sleep disorder, aches, urinary symptoms, thus severely affect quality of life (34–36). A study from Turkey showed that BMI is a significant independent factor for severity of menopause symptoms (37). WHO defined overweight as adults with BMI scores 25.0–29.9 kg/m² and obesity as BMI scores 30.0 kg/m² or higher. In China, the evidence based recommended BMI thresholds of overweight and obesity was adjusted to 24·0 kg/m² and 28·0 kg/m² (17). When at the same level of body mass, Chinese populations seem to have more visceral fat than White people (38). Because BMI is an indicator of general obesity, which fails to reflect central obesity, WHR has been integrated in the study as a more specific measure.

In this study, we found an increase of abdominal obesity in menopausal women with increasing age, which matches the outcome with some studies (13, 39). On the contrary, other researchers revealed an elevated risk of obesity in peri-/postmenopausal women compared with premenopausal women, independent of aging (40, 41).

Previous studies examining the association between moderate/severe VMS and BMI have reported conflicting findings (42). Some previous studies have demonstrated that higher BMI was related to more severe VMS (36, 43), which are consistent with our findings. We found a greater than two-fold increasing of abdominal obesity in those women who reported having moderate/severe bothered VMS compared with women not bothered by VMS. We found there is no significant relationship between low frequency or not bothering VMS and obesity or abdominal obesity. Central obesity is one of the manifestations of metabolic syndrome. The possible mechanisms may relate to the sympathetic overactivity that exists in both VMS and metabolic syndrome (44, 45). The decline of estrogen during menopause interferes the metabolism of neurotransmitters (e.g., serotonin, noradrenaline) thus leading to hot flashes by modulating the set point temperature (8, 46).

Consistent evidence showed that there seemed to be a bilateral relevance between overweight/obesity and emotional distress in normal population (47). In fact, adipokines produced by fatty tissues activate systemic inflammation, which affects the brain, leading to mood dysregulation (48). There are some possible assumptions that mood disorders related to menopause increase the generation of cytokines and free radicals, resulting in more fat deposition (9). Obesity would change the self- esteem of women, leading to mood disorders to some extent (49). Myint et al. found that an increase in WHR, but not in BMI, was significantly associated with lower mental health (50). However, related researches were quite limited and the correlation seems not strong (47, 51, 52). Our study showed that obese women are more likely to have depressive symptoms and less likely to have anxiety symptoms. However, the correlation between obesity and depressive symptoms was not statistically significant, which may be related to the small sample size. A strong relationship between VMS and mood symptoms during menopause has been observed (53, 54). Frequently/severe VMS leads to extreme emotional discomfort, and consequently influences life experience (55).

The ‘thermoregulatory model’ proposes that adiposity prevents heat dissipation, thus increasing the severity of menopausal symptoms (56, 57), which may explain the correlation between obesity and menopausal symptoms to some extent. Multi studies have shown that obesity contributes to estrogen deficiency (58). The underlying cause of menopause symptoms - the drop in estrogen levels - can enhance metabolic dysfunction and induce obesity (59–61). Our study demonstrated that higher BMI is related to severe VMS, supporting the ‘thermoregulatory model’ to some extent. Sexual symptoms gradually become more prominent with advancing age, which may be related to the gradual loss of sexual functioning and low estrogen levels. There exist some controversies on the relationship between obesity and sexual functioning. Some research has suggested that there is no link between BMI and female sexual dysfunction (49, 62–64). Other studies reported different results. Previous cross-sectional studies found no association between BMI level and sexual functioning without considering age and menopausal status. However, after controlling for covariates, there seems to be a positive correlation between higher BMI and lower frequency of intercourse (64, 65). A case–control study reported decreased female sexual function index scores correlated with overweight (66). The Study of Women’s Health Across the Nation found BMI was not associated with overall changes in sexual functioning, however, continuous extra weight gain was associated with reduced sexual desire and intercourse frequency (67). Our study suggested that sexual functioning symptoms were independently associated with obesity.

The present study has several strengths. First, this study is one of the most comprehensive studies to measure both the prevalence and the severity of VMS across four distinctive reproductive stages, using a validated instrument. Moreover, ours was one of few studies that investigated menopause symptoms and associated factors in Asian women using the MENQOL, a validated instrument for assessing quality of life. The strengths of this study are its long-term, prospective examination of symptoms in the transition to menopause that captures the early stages of the transition in late reproductive-age women in community rather than medical institution. Results from this study are more likely to be representative of urban women in China than those using samples from medical institution.

Study limitations also should be considered. First, the participants were followed-up annually, so short-term changes may not have been fully identified. Second, the evaluation of hot flashes frequency is based on participants’ self-reports, rather than in-depth assessments or diagnoses of the symptoms. The assessment of mood symptoms relies on the HADS questionnaire, which is not equivalent with clinical diagnosis of diseases, caution should be taken in to extrapolate the results. Third, we only included natural menopausal women without hormone treatment. Our results may not apply to women who undergo surgical menopause and hormone users.

In summary, the data indicated that obesity and abdominal obesity increased during menopause and was related to menopausal symptoms, including severe VMS and decreased anxiety symptoms. The obesity also associated with increased sexual functioning symptoms. We found the increase of abdominal obesity was more rapidly than obesity in midlife women. Appropriate measures should be taken to reduce the incidence of abdominal obesity, in order to prevent related complications and promote women’s health. The findings are important for better understanding the physical and physiologic changes that occur during the menopausal transition in Chinese women. Future studies are required to clarify the specific mechanism between obesity and menopause symptoms.

## Data Availability Statement

The raw data supporting the conclusions of this article will be made available by the authors, without undue reservation.

## Ethics Statement

The studies involving human participants were reviewed and approved by the institutional review board of the Peking Union Medical College Hospital. The patients/participants provided their written informed consent to participate in this study.

## Author Contributions

RT, YF, SL, and RC participated in the design of the study, analysis, interpretation of the data, and drafting and revising of the paper. ML, DZ, FH, ZX, and YCW participated in the design of the study, gathering of data, and writing of the manuscript. YPW provided critical revisions to improve intellectual content. GL participated in the design of the study and gathering and analysis of data. All authors conceptualized the paper and read and approved the final version of the manuscript.

## Funding

This work was supported by (1) National Key Research and Development Program (2018YFC1004801), (2) Chinese Academy of Medical Science Innovation Fund for Medical Sciences (CIFMS) (2020-I2M-CT-B-040), and (3) the National Natural Science Foundation of China Project (grant number 81871141).

## Conflict of Interest

The authors declare that the research was conducted in the absence of any commercial or financial relationships that could be construed as a potential conflict of interest.

## Publisher’s Note

All claims expressed in this article are solely those of the authors and do not necessarily represent those of their affiliated organizations, or those of the publisher, the editors and the reviewers. Any product that may be evaluated in this article, or claim that may be made by its manufacturer, is not guaranteed or endorsed by the publisher.
